# Blood Pressure Control in Traumatic Subdural Hematomas

**DOI:** 10.7759/cureus.30654

**Published:** 2022-10-25

**Authors:** David R Hallan

**Affiliations:** 1 Neurosurgery, Penn State Health Milton S. Hershey Medical Center, Hershey, USA

**Keywords:** map, sbp, tbi, sdh, mortality, outcomes, systolic blood pressure, blood pressure, traumatic brain injury, subdural hematoma

## Abstract

Background

There is debate over optimal systolic blood pressure (SBP) after traumatic subdural hematoma. Increased SBP has the benefit of increasing cerebral perfusion pressure and limiting the detrimental secondary effects of traumatic brain injury but poses a risk of hematoma expansion. While prior studies have shown that SBP<90mmHg is associated with worsened morbidity and mortality in subdural hematoma patients, clinical guidelines and expert opinion have differing initial SBP goals. The aim of this study is to leverage a large database to determine the effects of two such goals, namely SBP 100-150mmHg versus SBP<180mmHg in this patient population.

Methods

A de-identified database network (TriNetX Research Network) was used to retrospectively query all patients with a first instance diagnosis of acute traumatic SDH, who also had a recorded GCS, with maintenance of SBP 100-150 within the first 24 hours (cohort 1) versus patients with an SBP<180 (cohort 2). Data came from 68 health care organizations (HCOs) with a total of 105,897,964 patients on 9/1/2022. The primary outcome of interest was mortality within 30 days. Secondary outcomes include gastrostomy tube placement, craniotomy/craniectomy/burr hole drainage, venous thromboembolism, ischemic stroke, myocardial infarction, seizure, falls, cardiac arrest, and acute kidney injury within 30 days. Cohorts were propensity-score matched for confounders.

Results

After propensity score matching, 1,243 patients were identified in each cohort. Age at index was 57.97+/-23.21 years and 58.28+/-22.35 years for cohorts 1 and 2, respectively. Mortality was seen in 243 patients (19.756%) vs. 209 (16.992%) (OR 1.203, 95% CI (0.98,1.476), p=0.0767) in cohorts 1 and 2, respectively. There was no statistical difference in secondary outcomes.

Conclusion

The results of this study demonstrate that the primary outcome of mortality at 30 days is not statistically different in acute traumatic SDH patients, whether their SBP is kept at 100-150 or below 180. Likewise, it shows no statistical difference in the subsequent incidence of gastrostomy tube placement, craniotomy/craniectomy/burr holes, venous thromboembolism, ischemic stroke, myocardial infarction, seizure, falls, or acute kidney injury.

## Introduction

There is debate over optimal systolic blood pressure (SBP) after traumatic subdural hematoma (SDH) [1]. While hypotension, defined as SBP<90mmHg, is predictive of increased mortality in traumatic brain injury (TBI) patients [1-8], hypertension is associated with an increased risk of hematoma expansion [9-11], or the cause of the hematoma itself [12]. While Brain Trauma Foundation (BTF) fourth edition guidelines and expert opinion suggest SBP>100 for patients aged 50-69, SBP>110 for patients aged 15-49 or >70 years, and mean arterial pressure (MAP) >70 [2], Seattle International Brain Injury Consensus Conference recommendations are for SBP>90 and goal cerebral perfusion pressure (CPP) of 70 [5]. Attending physician preference at our institution (Penn State Milton S. Hershey Medical Center) for traumatic SDH blood pressure management in the absence of invasive intracranial monitoring has the propensity to gravitate towards one of two schools of management: keeping SBP 100-150 with MAP>65, or maintaining SBP<180 and MAP>65. The study aims to leverage an extensive database (TriNetX) to determine if there is a difference in 30-day outcomes in traumatic acute subdural hemorrhage patients who have maintained these two different SBP goals for at least 24 hours after their brain injury after propensity score matching for initial Glasgow coma score (GCS), and common comorbidities and risk factors.

## Materials and methods

A de-identified database network (TriNetX) was used to retrospectively query via International Classification of Disease (ICD-10) codes and Common Procedural Terminology (CPT) codes to evaluate all patients with a first instance diagnosis of acute traumatic SDH, who also had a recorded GCS, with the maintenance of SBP 100-150 within the first 24 hours (cohort 1) versus patients with an SBP<180 (cohort 2). Data came from 68 health care organizations (HCOs) with a total of 105,897,964 patients on 9/1/2022. Data includes demographics, diagnoses, medications, laboratory values, and procedures. The identity of the patients and HCOs is not disclosed to comply with ethical guidelines prohibiting data re-identification. An IRB waiver was granted. Previous literature informed our use of this database and its validity, and the network's exact details have been previously described [13-16].

The primary outcome of interest was mortality within 30 days. Secondary outcomes include gastrostomy tube placement, craniotomy/craniectomy/burr hole drainage, venous thromboembolism, ischemic stroke, myocardial infarction, seizure, falls, cardiac arrest, and acute kidney injury within 30 days. To adjust for hypothesized confounders on the relationship between SDH and the outcomes of interest, medical information, including age at the date of SDH, as well as sex, race, GCS scores 13-15, 9-12, and 3-8, and the comorbidities of traumatic subarachnoid hemorrhage, epidural hematoma, hypertension, obesity, ischemic heart disease, diabetes, chronic kidney disease, prior acute kidney failure, atrial fibrillation and flutter, smoking history, alcohol abuse, liver cirrhosis, and antiplatelet and anticoagulant medications was gathered. Analysis was performed using unmatched and propensity score-matched cohorts, with the greedy-nearest neighbor algorithm with a caliper of 0.1 pooled standard deviations. Chi-square analysis and logistic regression were performed on categorical variables.

## Results

After propensity score matching, 1,243 patients were identified in each cohort. Age at index was 57.97+/-23.21 years and 58.28+/-22.35 years for cohorts 1 and 2, respectively. 67.578% vs. 67.820% of patients were male. Average SBP was 125+/-19 mmHg vs. 159+/-30.1 mmHg. Initial GCS score, based on both GCS at emergency arrival and GCS at hospital admission, was approximately 13-15 for 59.131% of patients in cohort 1 and 59.936% in cohort 2, 9-12 for 8.287% vs. 7.964%, and 3-8 for 34.726% vs. 34.353%. Baseline demographics and characteristics, both pre- and post-matching are shown in Table 1.

**Table 1:** Baseline demographics and characteristics before and after propensity score matching

		Before Matching			After Matching		
Code	Diagnosis	Cohort 1, n (%)	Cohort 2, n (%)	Std diff.	Cohort 1, n (%)	Cohort 2, n (%)	Std diff.
AI	Age at Index	52.68+/-25.26 years	60.19+/-21.88 years	-	57.97+/-23.21 years	58.28+/-22.35 years	-
M	Male	1261 (67.003)	997 (68.334)	0.028465	840 (67.578)	843 (67.82)	0.0051612
F	Female	620 (32.944)	462 (31.666)	0.0273345	403 (32.422)	400 (32.18)	0.0051612
2106-3	White	1325 (70.404)	902 (61.823)	0.1820345	818 (65.809)	824 (66.291)	0.0101936
2131-1	Unknown Race	256 (13.603)	274 (18.78)	0.140898	206 (16.573)	201 (16.171)	0.0108713
2054-5	Black or African American	236 (12.54)	201 (13.777)	0.0365912	166 (13.355)	166 (13.355)	0
2028-9	Asian	22 (1.169)	17 (1.165)	0.0003527	14 (1.126)	16 (1.287)	0.0147366
R40.2412	Glasgow coma scale score 13-15, at arrival to emergency department	871 (46.281)	627 (42.975)	0.0665401	539 (43.363)	548 (44.087)	0.0145969
R40.2413	Glasgow coma scale score 13-15, at hospital admission	288 (15.303)	246 (16.861)	0.0424197	196 (15.768)	197 (15.849)	0.0022052
R40.2422	Glasgow coma scale score 9-12, at arrival to emergency department	108 (5.739)	100 (6.854)	0.0459343	77 (6.195)	76 (6.114)	0.0033476
R40.2423	Glasgow coma scale score 9-12, at hospital admission	42 (2.232)	25 (1.714)	0.0372694	26 (2.092)	23 (1.85)	0.0173637
R40.2432	Glasgow coma scale score 3-8, at arrival to emergency department	426 (22.635)	355 (24.332)	0.0400235	309 (24.859)	303 (24.377)	0.0112054
R40.2433	Glasgow coma scale score 3-8, at hospital admission	200 (10.627)	137 (9.39)	0.0412265	126 (10.137)	124 (9.976)	0.00535
I10-I16	Hypertensive diseases	824 (43.783)	901 (61.755)	0.3659574	709 (57.039)	718 (57.763)	0.0146428
S06.6	Traumatic subarachnoid hemorrhage	942 (50.053)	690 (47.293)	0.0552499	619 (49.799)	591 (47.546)	0.0450796
E78	Disorders of lipoprotein metabolism and other lipidemias	485 (25.77)	487 (33.379)	0.1672987	396 (31.858)	407 (32.743)	0.0189253
F17	Nicotine dependence	440 (23.379)	332 (22.755)	0.0148147	304 (24.457)	274 (22.043)	0.0571578
I20-I25	Ischemic heart diseases	357 (18.969)	321 (22.001)	0.0751828	280 (22.526)	283 (22.767)	0.0057665
E08-E13	Diabetes mellitus	310 (16.472)	346 (23.715)	0.1815031	264 (21.239)	271 (21.802)	0.0137035
N17-N19	Acute kidney failure and chronic kidney disease	335 (17.8)	324 (22.207)	0.1103295	264 (21.239)	266 (21.4)	0.0039286
Z87.891	Personal history of nicotine dependence	346 (18.385)	304 (20.836)	0.0617723	258 (20.756)	256 (20.595)	0.0039731
S06.3	Focal traumatic brain injury	420 (22.317)	275 (18.849)	0.08586	257 (20.676)	247 (19.871)	0.0200117
R13	Aphagia and dysphagia	317 (16.844)	322 (22.07)	0.1323054	245 (19.71)	243 (19.549)	0.0040509
R53	Malaise and fatigue	353 (18.757)	255 (17.478)	0.0332094	233 (18.745)	239 (19.228)	0.012308
J40-J47	Chronic lower respiratory diseases	313 (16.631)	247 (16.929)	0.0079788	218 (17.538)	221 (17.78)	0.0063294
I48	Atrial fibrillation and flutter	290 (15.409)	243 (16.655)	0.0339675	215 (17.297)	213 (17.136)	0.004262
F10.1	Alcohol abuse	304 (16.153)	224 (15.353)	0.0219626	201 (16.171)	188 (15.125)	0.0287902
S06.9	Unspecified intracranial injury	286 (15.197)	213 (14.599)	0.0167828	187 (15.044)	185 (14.883)	0.0045106
S06.1	Traumatic cerebral edema	269 (14.293)	215 (14.736)	0.0125713	183 (14.722)	177 (14.24)	0.013717
S06.2	Diffuse traumatic brain injury	259 (13.762)	172 (11.789)	0.059132	161 (12.953)	153 (12.309)	0.0193752
I50	Heart failure	205 (10.893)	167 (11.446)	0.0175736	150 (12.068)	150 (12.068)	0
E65-E68	Overweight, obesity and other hyperalimentation	188 (9.989)	177 (12.132)	0.0683414	146 (11.746)	139 (11.183)	0.0176772
R51	Headache	234 (12.434)	164 (11.241)	0.0369361	146 (11.746)	146 (11.746)	0
F10.2	Alcohol dependence	161 (8.555)	132 (9.047)	0.0173867	117 (9.413)	103 (8.286)	0.0396645
R63	Symptoms and signs concerning food and fluid intake	157 (8.342)	127 (8.705)	0.012979	109 (8.769)	107 (8.608)	0.0057124
S06.4	Epidural hemorrhage	181 (9.617)	94 (6.443)	0.1170189	84 (6.758)	89 (7.16)	0.0158089
I73	Other peripheral vascular diseases	93 (4.942)	81 (5.552)	0.0273698	69 (5.551)	71 (5.712)	0.0069797
K74	Fibrosis and cirrhosis of liver	51 (2.71)	45 (3.084)	0.0223249	39 (3.138)	37 (2.977)	0.0093465
1191	Aspirin	375 (19.926)	372 (25.497)	0.1332724	299 (24.055)	296 (23.813)	0.0056565
32968	Clopidogrel	100 (5.313)	102 (6.991)	0.0698588	80 (6.436)	84 (6.758)	0.0129642
11289	Warfarin	122 (6.482)	86 (5.894)	0.0244064	77 (6.195)	83 (6.677)	0.0196715
1364430	Apixaban	59 (3.135)	38 (2.605)	0.0317754	38 (3.057)	37 (2.977)	0.0047033
1114195	Rivaroxaban	35 (1.86)	28 (1.919)	0.0043627	28 (2.253)	26 (2.092)	0.0110379
31500	Intubation, endotracheal, emergency procedure	291 (15.462)	203 (13.914)	0.044	191 (15.366)	188 (15.125)	0.0067143

Mortality was seen in 243 patients (19.756%) vs. 209 (16.992%) (OR 1.203, 95% CI (0.98,1.476), p=0.0767) in cohorts 1 and 2, respectively. Gastrostomy tube was seen in 73 (5.945%) vs. 79 (6.397%) (OR 0.925, 95% CI (0.666,1.284), p=0.641) patients in cohorts 1 and 2, respectively. Craniotomy/Craniectomy/Burr holes was seen in 158 (12.711%) vs. 150 (12.068%) (OR 1.061, 95% CI (0.836,1.347), p=0.6263) patients in cohorts 1 and 2, respectively. Venous thromboembolism was seen in 90 (7.673%) vs. 74 (6.234%) (OR 1.25, 95% CI (0.909,1.719), p=0.1694). Ischemic stroke was seen in 63 (5.334%) vs. 74 (6.314%) (OR 0.836, 95% CI (0.591,1.182), p=0.3103). Myocardial infarction was seen in 26 (2.174%) vs. 23 (1.921%) (OR 1.134, 95% CI (0.643,1.999), p=0.6628). Seizure was seen in 158 (13.763%) vs. 161 (14.135%) (OR 0.969, 95% CI (0.765,1.228), p=0.7973). Falls was seen in 242 (24.08%) vs. 267 (25.165%) (OR 0.943, 95% CI (0.772,1.152), p=0.5672). Cardiac arrest was seen in 33 (2.696%) vs. 52 (4.221%) (OR 0.629, 95% CI (0.403,0.98), p=0.0387). Acute kidney injury was seen in 52 (4.183%) vs. 66 (5.31%) (OR 0.779, 95% CI (0.537,1.13), p=0.1867). Table 2 shows outcomes after propensity score matching.

**Table 2:** Outcomes after propensity score matching

Outcome	Cohort 1, n (%)	Cohort 2, n (%)	Odds ratio (95% CI)	95% CI	P-value
Deceased	243 (19.756)	209 (16.992)	1.203	(0.98,1.476)	0.0767
Gastrostomy tube	73 (5.945)	79 (6.397)	0.925	(0.666,1.284)	0.641
Craniotomy/Craniectomy/Burr holes	158 (12.711)	150 (12.068)	1.061	(0.836,1.347)	0.6263
Venous thromboembolism	90 (7.673)	74 (6.234)	1.25	(0.909,1.719)	0.1694
Ischemic stroke	63 (5.334)	74 (6.314)	0.836	(0.591,1.182)	0.3103
Myocardial infarction	26 (2.174)	23 (1.921)	1.134	(0.643,1.999)	0.6628
Seizure	158 (13.763)	161 (14.135)	0.969	(0.765,1.228)	0.7973
Falls	242 (24.08)	267 (25.165)	0.943	(0.772,1.152)	0.5672
Cardiac arrest	33 (2.696)	52 (4.221)	0.629	(0.403,0.98)	0.0387
Acute kidney injury	52 (4.183)	66 (5.31)	0.779	(0.537,1.13)	0.1867

## Discussion

The results of this study demonstrate that the primary outcome of mortality at 30 days is not statistically different in traumatic SDH patients, whether their SBP is kept at 100-150mmHg or below 180mmHg. Likewise, it shows no statistical difference in the subsequent incidence of gastrostomy tube placement, craniotomy/craniectomy/burr holes, venous thromboembolism, ischemic stroke, myocardial infarction, seizure, falls, or acute kidney injury.

TBI is a leading cause of death and disability, and SDH accounts for approximately 11% of TBI [5,11]. SDH management has room for improvement [11]. While the primary brain insult cannot be reversed, secondary damage can be limited [7]. Secondary brain injury can result from impaired cerebral blood flow, impairment of autoregulation, disruption of tissue oxygenation and brain metabolism, tissue inflammation and necrosis, edema, oxidative stress, and vasospasm, and from hematoma expansion due to factors such as hypertension [5,6,9-12]. On the other hand, any episode of hypotension, defined as SBP<90, has been associated with increased mortality and worsened neurological outcomes in severe TBI [1-8,17]. This could be due to the critical importance of maintaining optimal cerebral perfusion pressure, which can mitigate the detrimental effects of cerebrovascular autoregulation impairment in TBI patients, thus reducing ischemic and hyperemic brain damage [2-4,17-20]. While SBP goals have been established for intraparenchymal hemorrhage, it is less clear for subdural hematomas. Some guidelines and consensus/expert opinions suggest SBP>90, and others SBP>110 or SBP>115 [2,6]. There are no clear clinical trials that address a specifically targeted SBP in SDH [11,12].

In balancing the alteration of SBP goals to limit the progression of hematoma and hemorrhagic transformation and reducing hypoperfusion and secondary cerebral ischemic insult, prior studies provide some elucidation.

In 2021, Asmar et al. performed a retrospective review of the American College of Surgeons Trauma Quality Improvement Program database looking at adult patients with TBI, their SBP, and the primary outcome of in-hospital mortality. A subgroup analysis was likewise performed based on the GCS score. The authors found that of 94,411 TBI patients, the mean SBP was 147+-28, and overall mortality was 8.6%. They found that the lowest mortality was between SBP 110-149 and the highest mortality for SBP<90 and SBP>190. SBP 110-149 also had the lowest mortality for their GCS subgroup analysis [1].

The IMPACT prospective database looked at 8,172 TBI patients and found that SBP<120 was associated with poor neurological recovery [2,8].

Erickson et al. in 2021 published a study looking at MAP and discharge outcomes for pediatric patients with severe TBI. The authors examined 166 children (aged <18 years) with GCS<9. 20.4% of the patients had a poor discharge outcome (defined as in-hospital mortality or discharge to a skilled nursing facility or long-term care facility). This poor outcome was most common in patients who were <5th percentile for low MAPs in the first 12 hours and the second most common for patients with MAPs in the 5^th^-9^th^ percentile in the first 12 hours. Likewise, the authors found that SBP below the 5^th^ percentile in the first 12 hours was likewise associated with poor discharge outcomes. The authors concluded that low MAPs were more strongly associated with poor discharge outcomes for pediatric TBI patients than low SBP, noting that low SBP had a higher predictive ability [17].

In 2019, a paper by Heino et al. looked at risk factors for recurrent hematoma after surgery for acute traumatic SDH, with a total of 132 patients at one center in Finland. They retrospectively reviewed demographic, clinical, and laboratory data and found that patients with post-craniotomy hematoma (after evacuation of SDH) were more likely to have a lower maximum SBP. The mean maximal SBP in patients with post-craniotomy hematoma was 148 mmHg versus 160 mmHg in patients without post-craniotomy hematoma [21].

Kow et al. in 2020 examined the escalation of MAPs in severe TBI in 28 patients. They found that increasing MAPs to raise CPP was associated with a reduction in ICP in two-thirds of the cases, and an increase in ICP in one-third of the cases. This could be because an increase in MAP induces vasoconstriction; however, caution must be used in severe TBI patients due to dys-autoregulation. The authors found that the absence of hypotension at initial evaluation was associated with a lower ICP burden. The authors noted that prior studies have shown that in the majority of TBI patients, when MAP rises significantly, then ICP will fall, but that is not the case for all TBI patients. The authors concluded that the direction of ICP changes in the first 15 minutes after MAP increase can be useful to see if a MAP challenge is beneficial in guiding the management of intracranial hypertension [4].

A paper by Powers et al. in 2018 looked at factors associated with the progression of conservatively managed acute traumatic SDH. It followed 117 patients. The authors found that high systolic blood pressure, the presence of subarachnoid hemorrhage, and initial SDH volume were associated with changes in SDH size. Authors concluded that higher SBP was associated with increased expansion of SDH [11].

Looking at acceptable blood pressure levels in the prehospital setting for patients with traumatic brain injury, Shibahashi et al. examined a total of 34,175 adult patients from the Japan Trauma Data Bank. They found that an SBP<110 was associated with an OR 1.52 (95% CI 1.39-165) of in-hospital mortality [6].

For pediatric patients, Suttipongkaset et al. published a paper examining blood pressure thresholds and mortality in pediatric traumatic brain injury. The National Trauma Data Bank was queried for 10,473 children with severe TBI, with populations divided into percentile categories of SBP less than fifth percentile, SBP 5^th^-24^th^ percentile, 25^th^-74^th^ percentile, 75^th^ to 94^th^ percentile, and >=95^th^ percentile. The authors found that 2388 (22.8%) died while in the hospital, and that the relative risk of in-hospital mortality was 3.2 (95% CI 2.9-3.6) in the SBP <5th percentile, 2.3 (95% CI 2.0-2.7) in the 5^th^-24^th^ percentile, and 1.4 (95% CI 1.2-1.6) in the 25^th^ to 74^th^ percentile, as compared to the SBP 75^th^-94^th^ percentile group. The authors concluded that in children with severe TBI, SBP below the 75^th^ percentile was associated with increased risk of in-hospital mortality [22].

This study is not without limitations. The major limitation is that this study is retrospective. Furthermore, although GCS was able to be identified at the time of SDH, information was unable to be obtained on GCS at 30 days. Likewise, radiologic data was unavailable, and thus the degree of midline shift and size and location of SDH was unable to be identified. In addition, some degree of misidentification is always inevitable in large database studies.

## Conclusions

This retrospective cohort study compares acute traumatic SDH patients with initial management of SBP 100-150 mmHg versus less than 180 mmHg in the first 24 hours of brain injury. The results of this study demonstrate that the primary outcome of mortality at 30 days is not statistically different and similarly shows no statistical difference in the subsequent incidence of gastrostomy tube placement, craniotomy/craniectomy/burr holes, venous thromboembolism, ischemic stroke, myocardial infarction, seizure, falls, or acute kidney injury.
